# The effect of repair protocols and chewing simulation on the microtensile bond strength of two resin matrix ceramics to composite resin

**DOI:** 10.1186/s12903-024-03932-4

**Published:** 2024-02-02

**Authors:** Annan Ahmed Elkassaby, Mohamed M. Kandil, Ghada Atef Alian

**Affiliations:** Department of dental biomaterials, dentistry, Ain Shams University, Cairo, Egypt

**Keywords:** Polymer infiltrated ceramic network, Resin nanoceramics, Cyclic loading, Artificial aging, Mechanical fatigue, Mechanical aging

## Abstract

**Background:**

To assess the micro tensile bond strength (µTBS) of two resin matrix ceramic (RMC) blocks bonded to composite resin by using different repair protocols with and without chewing simulation (CS).

**Materials and methods:**

Two resin matrix ceramic blocks (Vita Enamic and Lava Ultimate) were divided into 4 groups according to the surface treatments: Bur grinding (control), Bur grinding + silane, 9.5% HF acid etching, and 9.5% HF acid etching + silane. The single bond universal adhesive was applied on all specimens after the surface treatments according to the manufacturer’s instructions, it was administered actively on the treated surface for 20 s and then light cured for 10 s, followed by incremental packing of composite resin to the treated surface. Each group was further divided into 2 subgroups (with/without chewing simulation for 500,000 cycles). A micro tensile bond strength test was performed for each group (*n* = 15). The effect of surface treatments on the materials was examined by using a scanning electron microscope (SEM). The micro tensile bond strength (MPa) data were analyzed with a three-way ANOVA, the independent t-test, and one-way ANOVA followed by the Tukey post-hoc test.

**Results:**

µTBS results were significantly higher for Lava Ultimate than Vita Enamic for all the surface treatment protocols with (*p* < 0.01). The chewing simulation significantly negatively affected the micro-tensile bond strength (*p* < 0.001). Bur grinding + saline exhibited the highest bond strength values for Lava Ultimate, both with and without chewing simulation. For Vita Enamic, bur grinding + saline and HF acid + saline showed significantly higher bond strength values compared to other surface treatments, both with and without chewing simulation (*p* ≤ 0.05).

**Conclusion:**

Bur grinding + silane could be recommended as a durable repair protocol for indirect resin matrix ceramics blocks with composite resin material.

## Introduction

Prefabricated computer-aided designing/ computer-aided manufacturing (CAD/CAM) blocks are becoming popular in clinical practice worldwide. They are used to produce a customized esthetic restoration that can provide great satisfaction for dental patients [1]. Resin matrix ceramic (RMC) CAD/CAM blocks successfully combined the merits of dental ceramics and composite resin [2]. Dental ceramics provide excellent esthetics, good biocompatibility, chemical inertness, and smooth surfaces that facilitate good gingival health [3, 4]. Dental composites are renowned for their favorable modulus of elasticity and decreased surface hardness [5]. Vita Enamic (VE) and lava ultimate (LU) are commercially available RMC blocks. VE (Vita Zahnfabrik, Bad Säckingen, Germany) is a polymer-infiltrated ceramic network made of a porous network of pre-sintered ceramics (86% by volume) infiltrated with a polymeric material, resulting in the integration of ceramics and polymers [6]. LU (3 M ESPE, St Paul, MN, USA) is commonly known as resin nanoceramics [5]. It is comprised of 80 wt% nanoceramic fillers and 20 wt%. polymeric resin [7]. However, ceramic restorations are highly prone to fracture [2]. So, to fix a chipped ceramic restoration, there are two treatment modalities, either to replace the whole restoration or to repair it intraorally using composite resin. The latter option is considered simpler, cheaper, and more practical to apply with a high success rate [8]. 

RMC blocks are formed by polymerization under controlled conditions of high pressure and temperature [9–11]. They exhibit a high degree of conversion with a reduced residual monomer content, rare carbon-carbon double bonds on the surface, together with the absence of the oxygen-inhibited layer. Thus, challenges are encountered to achieve durable repair of RMC CAD/CAM blocks with resin composite [10, 12, 13]. In the literature, several surface treatment protocols have been proposed to improve the durability of ceramic/composite resin bonds: including acid etching, airborne particle abrasion, and diamond bur grinding [1, 14–16]. 

Multiple studies were carried out to investigate the effect of aging, whether water storage or thermocycling, on these surface treatments [12, 17–20]. However, there were no sufficient studies to investigate the impact of mechanical stresses applied during mastication on the repaired restorations as the stresses become magnified at the bonded interface due to the mismatch in the elastic modulus between the two materials [17]. Therefore, there is no conclusive recommendation yet, that favors a durable repair protocol to be implemented to repair fractured RMC block restorations. According to the survey, the repair or replacement of a defective restoration performed by the American Dental Association Clinical Evaluators Panel, the most used surface treatments to repair all-ceramic restorations; are diamond bur, hydrofluoric acid, and silane coupling agents [21]. 

For the given reasons, this study aims to evaluate the bond strength of RMC CAD/CAM blocks bonded to composite resin, using different surface treatments with and without CS. The first null hypothesis is that the proposed surface treatments will not affect the micro tensile bond strength (µTBS) of the bonded RMC blocks. The second null hypothesis, there is no difference between the 2 different RMC blocks on the µTBS of the bonded RMC blocks. The third null hypothesis is that CS will not affect µTBS of the bonded RMC blocks.

## Materials and methods

The Materials used in this study are illustrated in Table 1.

**Table 1:** Materials used in the study, their brand names, compositions, manufacturers, and lot numbers

Material	Brand name	Composition	Manufacturer	Lot number
Polymer infiltrated ceramic	VITA Enamic	Polymeric matrix 14 wt% (UDMA, TEGDMA) Fillers 86 wt%: SiO2 (58–63%), Al2O3 (20–23%), Na2O (9–11%), K2O (4–6%), B2O3 & ZrO2 (< 2%). CAD/CAM block size (12 × 14 × 18 mm).	Vita Zahnfabrik, Bad Säckingen, Germany	55,313
Resin nanoceramic	Lava Ultimate	Bis-GMA, UDMA, Bis-EMA, TEGDMA, SiO2 (20 nm), ZrO2 (4–11 nm), ZrO2/SiO2 clusters, filler mass (80 wt%). CAD/CAM block size (12 × 14 × 18 mm).	3 M ESPE, St Paul, MN, USA	N770935
Universal adhesive	Single bond	10 Methacryloyloxydecyl dihydrogen phosphate, HEMA, silane, dimethacrylate resins, Vitrebond, copolymer, filler, ethanol, water, initiators	3 M ESPE, St Paul, MN, USA	5,695,133
Nanohybrid composite resin	Brilliant NG	Methacrylates, dented glass, and amorphous silica, with filler content of 80 wt%	Coltene, Altstätten, Switzerland	162,456
Hydrofluoric acid etchant	Porcelain etchant	9.5% buffered hydrofluoric acid gel	Bisco, Irving Park Rd. Schaumburg, IL, USA	2,000,001,191
Silane coupling agent	Porcelain primer pre-hydrolyzed silane primer	γ-methacryloxy-propyl-trimethoxy silane, ethanol, acetone	Bisco, Irving Park Rd. Schaumburg, IL, USA	2,000,001,245

**Fig. 1:**
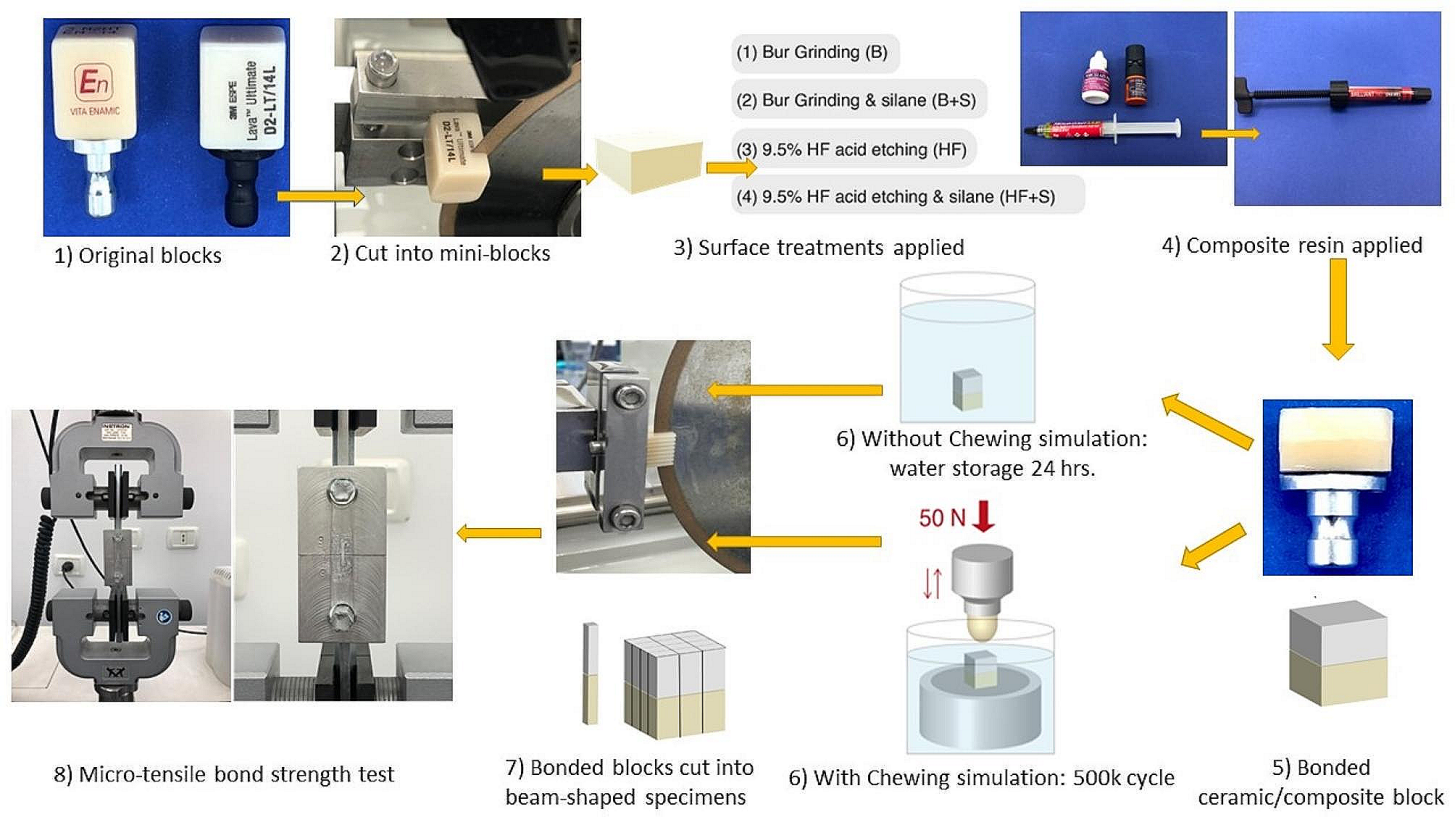
Workflow diagram for specimens’ preparation

The sample size calculated for the µTBS test was based on the data obtained from an internal pilot study using G*Power version 3.1.9.2 for sample size analysis at α = 0.05 and 80% power and effect size equal to 0.5202 that yields a sample size of 12 samples per group. Fifteen specimens per group were used to gain extra power. A total of 240 RMC/composite beam-shaped specimens were prepared for bond strength testing. The specimens were divided into 2 main groups according to the type of RMC block (VE and LU). Each material group was further subdivided into 4 subgroups according to the surface treatments. Each surface treatment group was divided into 2 groups (with and without CS) (*n* = 15). The original VE and LU blocks were cut using a 7-inch low concentration (LC) diamond wafer blade (Kemet, Maidstone, UK), mounted in a low-speed linear precision cutting saw (Isomet 4000, Buehler, Lake Bluff, IL, USA) into 8 mini-blocks (5 × 12 × 14 mm) from each material. The cutting procedure was performed at 3200 rpm and a feed rate of 6 mm/min., under copious water coolant. For each mini-block, the surface that will receive the surface treatment was wet-polished with 600, 800, 1000, and 1200 grit silicon carbide papers, respectively. Polishing was done in a unidirectional circular motion for 1 min, with light pressure to ensure a standardized surface roughness before applying the surface treatment on the polished surface. After that, all the polished blocks were cleaned ultrasonically in distilled water for 5 min; to ensure a clean, non-contaminated surface [22]. The polished blocks from each RMC block were randomly allocated into 4 subgroups and subjected to different surface treatments. (Fig. 1).

Bur grinding group (B) (Control): the polished surface was roughened with a standard wheel stone (Shenzhen Dian Fong Abrasives, Guangdong, China) for five strokes in one direction with light pressure, using a water-cooled high-speed handpiece [14]. 

Bur grinding + silane group (B + S): the same procedure as the B group [14]. Then, the surface was air-dried, and the silane coupling agent was applied for 60 s, and then gently dried for 5 s [4]. 

Hydrofluoric acid etching group (HF): the polished surface was subjected to 9.5% HF acid for 60 s, followed by rinsing for 60 s using an air-water spray [6]. 

Hydrofluoric acid etching + saline group (HF + S): the same procedure as the HF group, after drying the surface, the silane coupling agent was applied for 60 s, followed by gentle dryness for 5 s.

Following the surface treatments, the universal adhesive was applied according to the manufacturer’s instructions. It was administered actively on the treated surface for 20 s. Then lightly air-dried with air-water spray until no movement of the adhesive layer was visible to ensure that the solvent had evaporated. The universal adhesive was cured for 10 s for 4 overlapping cycles to cover the entire treated surface.

Afterward, the nanohybrid composite build-up was bonded to the surface-treated RMC blocks [23]. Putty impression mold (5 mm height×12 mm width×14 mm length) was used through which increments of 2 mm composite resin were packed on the treated surface and light-cured for 40 s for 4 overlapping cycles, using an LED light-curing unit with an output intensity of 1200 mW/cm^2^ (Elipar, S10, 3 M ESPE, Germany). Incremental packing of composite resin continued till the composite build-up reached the (5 mm height×12 mm width×14 mm length) [4]. Extra curing was done on the sides of the block against the celluloid matrix to ensure adequate curing.

Each RMC/composite block was divided into 2 groups: according to mechanical aging. In the first group (without CS), the ceramic/composite blocks were stored in distilled water for 24 h after bonding inside the incubator (Titanox, art. a3-213-400I, Italy) at 37 degrees Celsius. In the second group (with CS), the ceramic/composite blocks were subjected to 500,000 cycles in the chewing simulator (SD mechatronik CS-4, Germany). The ceramic/composite blocks were mounted inside the chewing simulator chambers, stabilized with an acrylic base, and submerged in distilled water. A ceramic ball was used as the antagonist, applying the 50 N force [24] vertically on the composite side of the bonded block with a frequency of 1.85 Hz.

The ceramic/composite blocks from all groups were sectioned longitudinally in two perpendicular directions by using the Isomet 4000. The Isomet cut the block in one longitudinal X-axis, and then the block was rotated 90° to produce the second cut in a longitudinal Y-axis. Both longitudinal X and Y directions are perpendicular to the bonded interface; [20] to create beam-shaped specimens (ceramic/composite) of approximately (0.9 × 0.9 × 10 mm). The samples from the edges of the blocks were excluded.

For µTBS testing, the beams were glued from their edges to the upper and the lower parts of special jigs using cyanoacrylate so that the bonded interface was centralized between the proximal jigs [25]. The specimens were tested in a universal testing machine (Instron 3356, UK) at 0.5 mm/min crosshead speed till fracture [20]. Bond strength values in MPa were calculated by Bluehill 3 software by dividing the maximum load at fracture by the surface area.

For Failure mode analysis, the fractured beams obtained from the µTBS test were examined using a digital microscope (Dino-lite pro) with 50× magnification power. The failure modes were classified into three categories: A-adhesive failure at the interface between the ceramic substrate and the composite resin, C-cohesive failure in the ceramic or the composite, and M-mixed failure involving both the adhesive and cohesive failure.

For SEM, three representative specimens for each material were examined with the SEM (*n* = 6). Each material received three surface pretreatments: no surface treatment, bur grinding, and HF acid etching to evaluate the morphological differences in the surface topographies induced by the proposed mechanical surface treatments. The specimens were mounted on metallic stubs and sputter-coated with gold using a sputter coater (Quorum, Q150T ES), then were examined using SEM (TEGSCAN, VEGA 3) at 1000x magnification power.

Statistical analysis was computed by using SPSS (statistical package for social sciences, IBM SPSS Statistics for Mac, version 24 software, Armonk, NY: IBM Corp, USA). The results were presented as means and standard deviations. The data were checked for normality by using the Kolmogorov – Smirnov test and the Shapiro test, and the results were normally distributed. Three-way ANOVA was carried out to explore the effect of the material, surface treatment, and aging on the micro tensile bond strength. Following significant interactions, an independent t-test was conducted to explore the effect of material and aging on the micro tensile bond strength. One-way ANOVA between groups was conducted to explore the effect of different surface treatments on micro tensile bond strength. Post-hoc comparisons using the Tukey test were used to investigate differences between groups, and the significance level was set at (*p* ≤ 0.05).

## Results

The means and standard deviation of µTBS testing are shown in Table 2.

**Table 2:** Mean values and standard deviation of micro tensile bond strength (µTBS) test (MPa)

Aging	Surface treatments	Vita Enamic (VE)	Lava Ultimate (LU)
Without chewing simulation	B	30.83 ± 4.04 ^Bb*^	44.84 ± 4.99 ^Abc*^
	**B + S**	44.26 ± 5.55 ^Ba*^	55.98 ± 5.72 ^Aa*^
	**HF**	26.80 ± 2.81 ^Bc*^	40.07 ± 5.37 ^Ac*^
	**HF + S**	42.28 ± 3.40 ^Ba*^	47.48 ± 4.55 ^Ab*^
**With chewing simulation**	**B**	21.25 ± 2.36 ^Bb^	35.37 ± 4.53 ^Aab^
	**B + S**	29.88 ± 3.89 ^Ba^	39.43 ± 5.60 ^Aa^
	**HF**	20.89 ± 2.83 ^Bb^	32.14 ± 4.91 ^Ab^
	**HF + S**	29.27 ± 2.72 ^Ba^	34.27 ± 5.34 ^Ab^

Different uppercase letters within the same row indicate significant differences between the RMC blocks. Different lowercase letters within the same column indicate significant differences between different surface treatments within each aging group. The presence of * within the same column indicates significant differences between the with and without chewing simulation groups

**Fig. 2:**
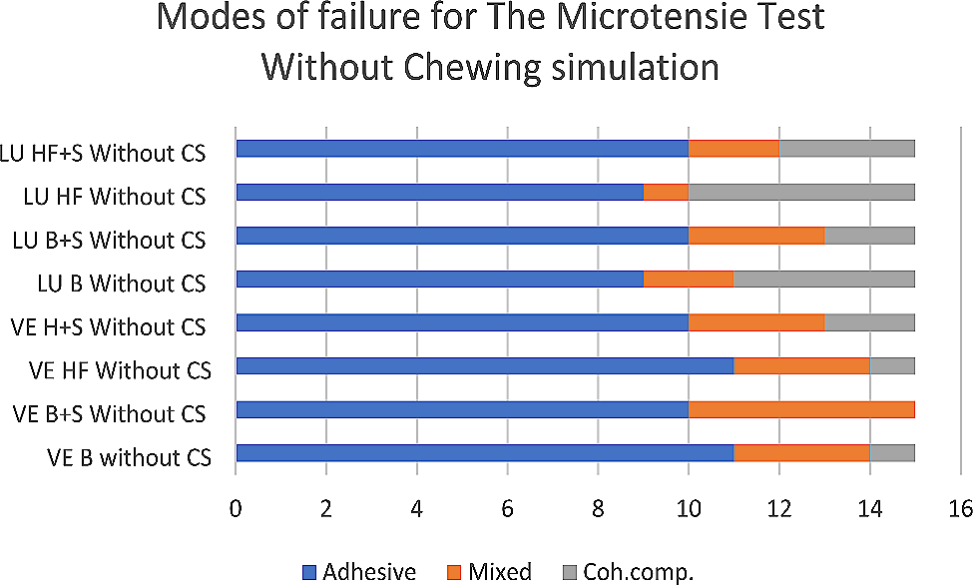
Different modes of failure for without chewing simulation groups

**Fig. 3:**
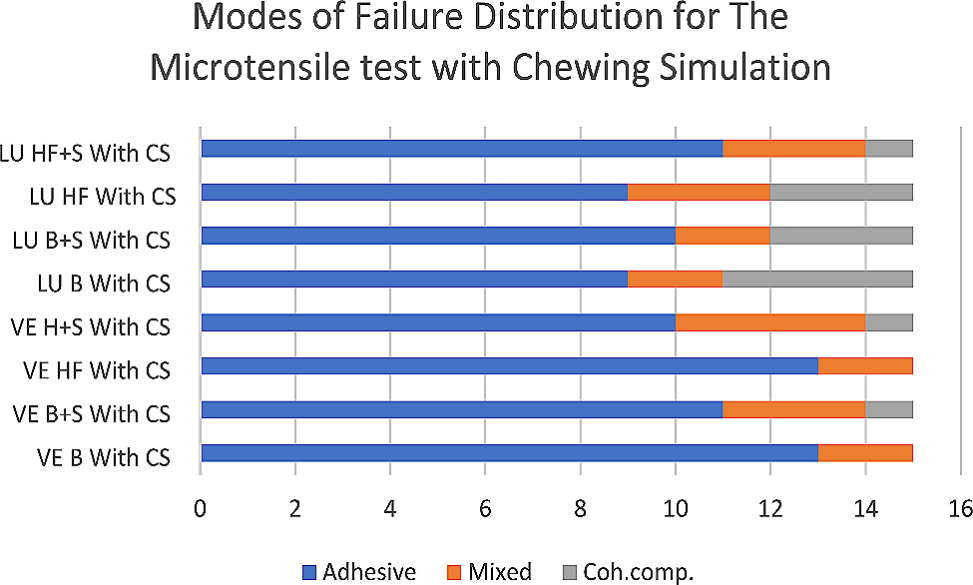
Different modes of failure for chewing simulation groups

For all groups, the T-test results for independent samples indicated that the (LU) groups showed significantly higher mean bond strength values compared to the (VE) groups (*p* < 0.01), irrespective of the effect of CS. For all groups, the T-test for independent samples indicated that (without CS) groups showed a significantly higher mean bond strength compared to (with CS) groups (*p* < 0.001), irrespective of the effect of the material and the different surface treatments. Then, the data were analyzed with One-way ANOVA, followed by the Tukey post hoc test. Failure modes distribution for the µTBS test is illustrated in Figs. 2 and 3.

**Fig. 4:**
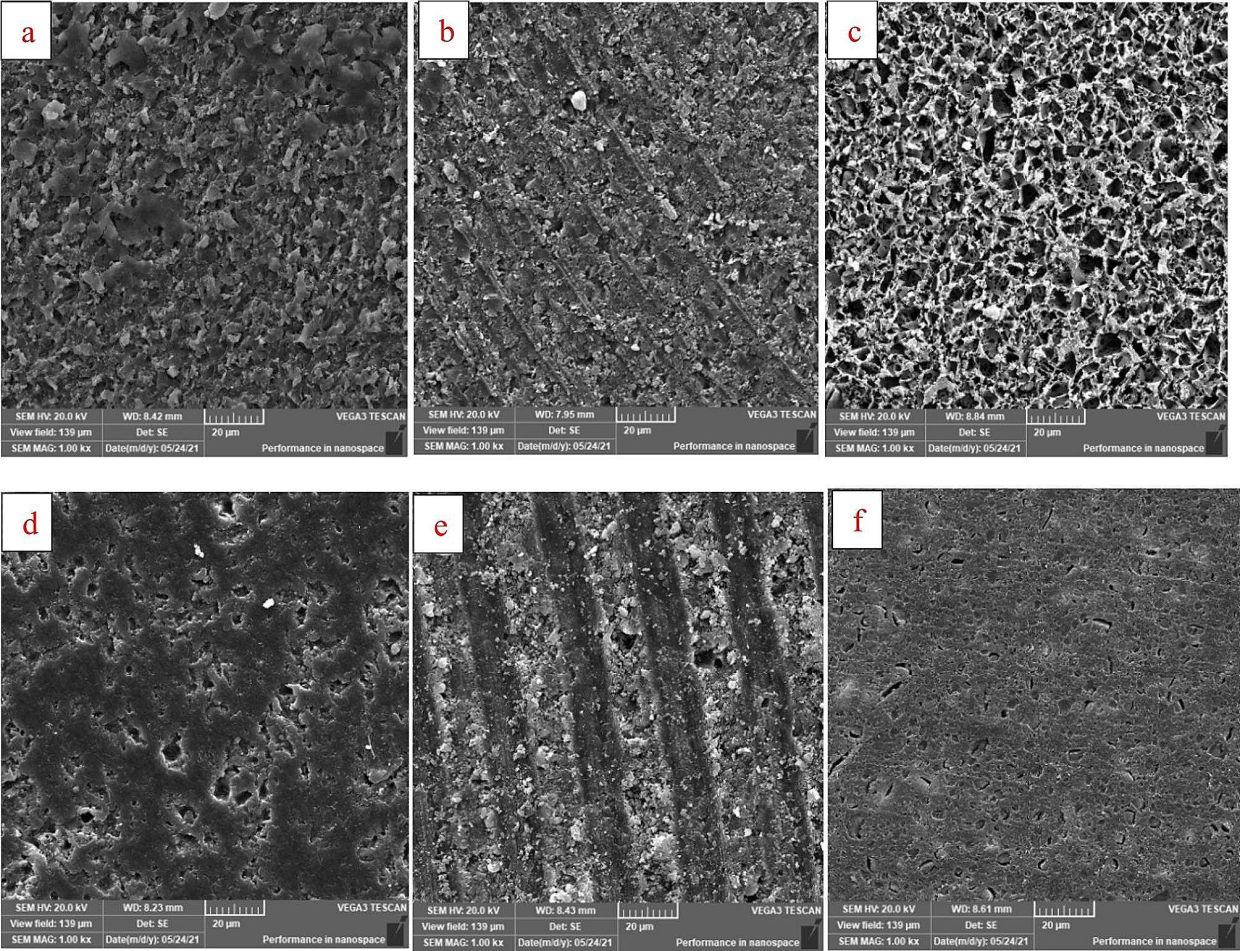
shows the SEM images at 1000x of (**a**) VE without surface treatment (**b**) VE with bur grinding (**c**) VE with HF acid etching (**d**) LU without surface treatment (**e**) LU with bur grinding (**f**) LU with HF acid etching

SEM images (Fig. 4) show the effect of the HF acid etching and Bur roughening surface treatments on the materials revealing a honeycomb-like structure formed by the remaining polymeric network after HF acid etching. However, LU showed different size porosities on the surface that were shallower, more irregular, and more in number in comparison to the no-treatment specimens. After bur roughening, both materials exhibited a ruffled surface topography that was more obvious on the LU with well-defined elevations and depressions.

## Discussion

In the present study, all null hypotheses were rejected as there was a significant difference between the surface treatments and their effect on the two RMC blocks. There was a significant difference between the two RMC blocks. Also, a significant decrease in the repaired bond strength values after CS was observed.

The surface treatments applied in this study were chosen due to their availability in dental clinics, ease of administration, and popularity of use in dental practice [21]. The choice of the diamond burs in this research as a mechanical surface treatment to boost the bond is because of its simplicity and cost-effectiveness in inducing surface roughness on the ceramic substrate [26]. Furthermore, it would be essential to refresh the material surface for bonding and remove the contaminated chipped surface layer of the restoration exposed to saliva. HF etching is the benchmark for ceramics as the HF acid dissolves the glassy phase leaving the crystalline phase, providing a porous adherend [19, 27, 28]. HF acid is often used to prepare the silicate-based ceramic surface for cementation extra-orally and intraoral repair. Despite being classified in 1998 as a highly hazardous chemical, its use becomes preferable when compared with additional removal of the tooth structure. However, rubber dam isolation is mandatory in this situation to avoid soft tissue contact and saliva contamination [29]. 

Airborne particle abrasion proved efficient in bond improvement. However, it had many shortcomings, including contamination of the surface with sand particles, risk of health complications, excessive volume loss from the treated surface [30], the possibility of material weakening due to crack formation, [31] and the costly device [15]. 

LU generally showed significantly higher bond strength values than VE for the µTBS results. This could be attributed to the difference in the elastic modulus and chemical composition between the VE and LU. The elastic modulus of substrates is a notable factor in determining bonding strength values. For the low young’s modulus materials, stresses disseminate throughout the material. While in the materials of high elastic modulus, stresses concentrate at the bonding interface [17]. A previous study stated that VE has an elastic modulus of almost three times the elastic modulus of LU [32]. This gives LU a superior advantage over VE in assessing the repair bond strength [17]. 

For VE, HF acid etching managed to offer a high repair bond strength comparable to Bur roughening. Regarding LU, Bur roughening provided superior bond strength results than HF acid etching. This difference could be attributed to the marked difference in the composition between those materials [33]. VE has a high portion of silica-based ceramic that can react with the HF acid, dissolving the glassy phase. The etching leaves the polymer network unchanged, creating a honeycomb-like appearance, and a high tendency for micromechanical interlocking [33]. However, LU contains mainly zirconia and zirconia-silica clusters which are more resistant to etching [18]. These were evident in the SEM images illustrating the effect of each surface treatment on the materials’ surface topography. On the contrary, the bur grinding induced a ruffled surface on both materials, enhancing their mechanical interlocking.

This study revealed that the additional use of the silane coupling agent after mechanical surface conditioning had increased the µTBS for VE with and without CS. This finding agrees with previous studies and literature [6, 16, 31, 34]. This outcome is most likely because of the glassy portion in the VE structure, where the silane coupling agent can chemically bond, unlike LU [32]. Therefore, it could be recommended to add a silane coupling agent to the repair protocol of RMC CAD/CAM blocks with composite resin [16]. 

CS was carried out for 500,000 cycles which is equivalent to 2 years of service [35]. The results of the two RMC blocks showed a significant reduction in the µTBS. Cyclic loading may have initiated microscopic cracks in areas of intense loading that propagated and coalesced with preexisting flaws, thus weakening the materials. [36] The results of our research agreed with the study performed by Al-Harbi et al. [36] Moreover, comparing the effect of silane application on the µTBS values for both materials with and without aging indicated that the chemical bond was negatively affected by aging. This reduction could be due to the susceptibility of the siloxane bond (Si-O-Si) to hydrolysis [33]. Despite the reduced bond strength values after aging, all proposed surface treatments produced bond strength values that exceeded the acceptable range of bond strength 15 to 25 MPa indicated for clinical situations [1]. Thus, they were proven to be durable, efficient, and practical, thus can be readily used to repair RMC blocks.

One of the limitations of this study is that only two mechanical surface treatments were used. Another limitation is that only the effect of masticatory forces was investigated, without taking into consideration other intraoral factors that can affect the bond strength including temperature fluctuations and pH changes.

## Conclusion

With the limitations in this study, it could be concluded that; the surface roughness induced by the bur grinding coupled with the separate silane coupling agent application was the most efficient durable repair protocol for both materials. Thus, it could be used instead of HF acid, which is hazardous and should be used with extreme caution intraorally.

## Data Availability

The datasets used and/or analyzed during the current study are available from the corresponding author upon reasonable request.

## Abbreviations

µTBS: Micro tensile bond strength
RMC: Resin matrix ceramic
CS: Chewing simulation
SEM: Scanning electron microscope
CAD/CAM: Computer-aided designing/ computer-aided manufacturing
VE: Vita Enamic
LU: Lava ultimate
LC: Low concentration
B: Bur grinding group
B + S: Bur grinding + silane group
HF: Hydrofluoric acid etching group
HF + S: Hydrofluoric acid etching + saline group
